# Tan Spots

**Published:** 1891-08

**Authors:** 


### TAN SPOTS.

Under various names attention has been directed from time immemorial to tanning and bronzing of the skin. So far as the discolorations are due to sunburn, the only sure way of avoiding them is to keep oneself in the dark, as if one were a photographic plate which must not be developed. The success of the numerous safeguards against sunburn is in direct proportion to their power of shading off the sunshine ; and it may be doubted if any thing yet discovered can beat the palm oil and ochre with which tropical savages delighted to besmear themselves ; and next I would place the hazeline and violet powder, which lies thick on people's cheeks at fashionable watering places, and is merely the civilized equivalent of the oil and ochre. Let the face be moistened with hazeline, or elder-flower water, and, when nearly dry, dusted with violet powder or vinolia powder, which must be removed where superfluous, and a certain amount of mitigation of the sunburn is sure to follow. But if the tan and freckles do appear, they can still be removed in much the same way as ink or iron moulds can be removed from linen. But it is scarcely worth while to attack discoloration alone, which is not accompanied with discomfort; for a berry-brown skin is not unsightly ; a vote of the human race would probably be preponderatingly in favor of a tan complexion—at all events, it is asserted that brown races never grow reconciled to the pallor of pale faces ; whereas, on the contrary, when white men have to live among dark-skinned races, they not only grow accustomed to the brown complexion, but even prefer it to their own. So we white people are not of the right color after all. However, as "Dot" prefers to remain white during the coming summer, she may use the violet powder, assisted by whatever protection in the way of bonnets and sunshades the current fashion may allow. Of all the prescriptions for removing the sunburn (if once got), rubbing the face with a lemon and cucumber parings is, perhaps, the simplest and best. To remove the discomfort of sunburn, as distinct from its stain, hazeline is good, and so is elder-water, and glycerine and cucumber—in fact, most of the lotions sold for the purpose—and they are numerous—have something in their favor.—*Ex.*

